# Targeting of Protein’s Messenger RNA for Viral Replication, Assembly and Release in SARS-CoV-2 Using Whole Genomic Data From South Africa: Therapeutic Potentials of *Cannabis Sativa* L

**DOI:** 10.3389/fphar.2021.736511

**Published:** 2021-09-02

**Authors:** Ochuko L. Erukainure, Motlalepula G. Matsabisa, Aliyu Muhammad, Musa M. Abarshi, James F. Amaku, Sanusi B. Katsayal, Adeline Lum Nde

**Affiliations:** ^1^ Department of Pharmacology, School of Clinical Medicine, Faculty of Health Sciences, University of the Free State, Bloemfontein, South Africa; ^2^ Department of Biochemistry, Faculty of Life Sciences, Ahmadu Bello University, Zaria, Nigeria; ^3^ Department of Chemistry, Michael Okpara University of Agriculture, Umudike, Nigeria

**Keywords:** Cannabis sativa, COVID-19, SARS-CoV-2, phytocannabinoids, South Africa

## Abstract

The possible evolutionary trend of COVID-19 in South Africa was investigated by comparing the genome of SARS-CoV-2 isolated from a patient in KwaZulu-Natal, South Africa with those isolated from China, Spain, Italy, and United States, as well as the genomes of Bat SARS CoV, Middle East Respiratory Syndrome Coronavirus (MERS-CoV), Mouse Hepatitis Virus (MHV), and Infectious Bronchitis Virus (IBV). Phylogenetic analysis revealed a strong homology (96%) between the genomes of SARS-CoV-2 isolated from KwaZulu-Natal, South Africa and those isolated from the study countries as well as those isolated from bat SARS CoV, MERS-CoV, MHV and IBV. The ability of phytocannabinoids from *Cannabis sativa* infusion to interact with gene segments (mRNAs) coding for proteins implicated in viral replication, assembly and release were also investiagted using computational tools. Hot water infusion of *C. sativa* leaves was freeze-dried and subjected to Gas Chromatography-Mass Spectroscopy analysis which revealed the presence of tetrahydrocannabivarin, cannabispiran, cannabidiol tetrahydrocannabinol, cannabigerol, and cannabinol. Molecular docking analysis revealed strong binding affinities and interactions between the phytocannabinoids and codon mRNAs for ORF1ab, Surface glycoprotein, Envelope protein and Nucleocapsid phosphoprotein from SARS-CoV-2 whole genome which may be due to chemico-biological interactions as a result of nucleophilic/electrophilic attacks between viral nucleotides and cannabinoids. These results depict the spread of SARS-CoV-2 is intercontinental and might have evolved from other coronaviruses. The results also portray the phytocannabinoids of *C. sativa* infusion as potential therapies against COVID-19 as depicted by their ability to molecularly interact with codon mRNAs of proteins implicated in the replication, translation, assembly, and release of SARS-CoV-2. However, further studies are needed to verify these activities in pre-clinical and clinical studies.

## Introduction

The outbreak of the corona virus disease 2019 (COVID-19) pandemic has led to an unprecedented pressure on the global health sector, with the sub-Saharan African countries as well as other developing countries being among the most hit (Chen et al., 2020; Willan et al., 2020). The disease which originated from Wuhan China in December 2019 as cluster crises of pneumonia, is caused by the novel β-coronavirus, Severe Acute Respiratory Syndrome Coronavirus 2 popularly regarded as SARS-CoV-2.

Italy, the United Kingdom and Spain were the epicenters for the pandemic in Europe, United States for North America, Brazil for South America, and South Africa for Africa. Although the cases of COVID-19 and mortality rate is lower in African countries compared to other continents, the pandemic has caused a lot of pressure on the poor health systems and economies of these countries which were already burdened with existing non-communicable diseases particularly malaria, AIDS and tuberculosis (Hogan et al., 2020; Sands, 2020; Sherrard-Smith et al., 2020). These setbacks coupled with an uncontrolled growing population exacerbates the continent’s vulnerability to the pandemic with major consequences. Egypt was the first to announce a case in the continent on February 14, 2020, this was followed by Nigeria on February 27, 2020, while South Africa announced her index case on March 5, 2020 (Lone and Ahmad, 2020; Zhao et al., 2020). These figures have so far grown to over 6 digits with South Africa presently having the highest cases. It is pertinent to mention that the virus was brought into the continent by travelers from Europe and has so far led to community spread.

The absence of a substantial standard protocol treatment and vaccine for the disease have led to continuous efforts by scientists and policy makers to curb the disease spread through mainly non-pharmaceutical interventions. These efforts include public sensitization on the virus and the disease, testing, patient isolation and contact tracing, as well as implementation of government policing such as national lockdown, social and physical distancing, mandatory public use of face mask, and constant washing of hands (Mitjà and Clotet, 2020). Despite South Africa passing some of the world’s stringent lockdown rules to curb the transmission of the virus, it has experienced an exacerbated growth in the number of cases which have caused tremendous strain on her health care system and economy. This has led to the search of novel and cheaper alternatives for the treatment of the diseases, with much attention given to indigenous medicinal plants. This has also led to the African stance through the WHO afro, Africa-CDC and the AU Commission in setting up an expert committee on traditional medicines for COVID-19 which aims at strengthening African countries by finding traditional medicines for the disease.

Several studies have reported the potentials of medicinal plants in the treatment and management of COVID-19 and its complications (Benarba and Pandiella, 2020; Erukainure et al., 2020; Vellingiri et al., 2020). These therapeutic properties have been attributed to the phytochemical constituents of the plants. Amongst these plants is *Cannabis sativa.*

*Cannabis sativa* is a member of the *Cannabis* genus and the Cannabaceae family. It is an annual herbaceous plant commonly known as weed, marijuana, Indian hemp, weed and dagga in different parts of the world. Its utilization cuts across religious and recreational purposes, as well as traditional medicine and food (Kuddus et al., 2013; Bonini et al., 2018). The plant is now the most reported for its pharmaceutical medical uses and it is regarded as a global specie as it is widely distributed across the world (UNODC, 2012). Although its utilization is under strict rules in most countries, *C. sativa* has over the years been reported to be effective in the treatment and management of several ailments such as diabetes mellitus (Ren et al., 2016), different cancer types (Guzman, 2003), insomnia (Ramar et al., 2018), epilepsy (Fusar-Poli et al., 2009), severe pains (Whiting et al., 2015) and neurodegeneration (Aso and Ferrer, 2016). These medicinal properties have been attributed to the phytochemical constituents of *C. sativa*, particularly the phytocannabinoids. Cannabidiol (CBD), tetrahydrocannabinol (THC) and cannabinol (CBN) constitute the predominant phytocannabinoids in *C. sativa* (Andre et al., 2016), and have been studied widely for their medicinal applications.

Recently, *C. sativa* has been gaining interests as a possible therapy for the treatment of COVID-19 and its complications (Byrareddy and Mohan, 2020; Mabou Tagne et al., 2020). This has been attributed to the antiviral activities of its phytocannabinoids such as CBD against hepatitis C and Kaposi sarcoma (Mabou Tagne et al., 2020), as well as its potent anti-inflammatory properties as depicted by its ability to suppress the production of TNF-α, MIP-1a, IL-2, 6, 1α and 1β, MCP-1, and interferon gamma (Nichols and Kaplan, 2020). These proinflammatory cytokines have been implicated in the pathogenesis and progression of COVID-19 induced by SARS-CoV-2 via the cytokine release syndrome (CRS) (Mehta et al., 2020; Nichols and Kaplan, 2020; Shi et al., 2020) commonly regarded as the cytokine storm. Therefore, studying the potentials of *C. sativa* and its phytocannabinoids as a possible therapy in the treatment and management of COVID-19 will contribute to the curbing of the disease.

This study aimed at decoding the possible evolutionary trend of COVID-19 in South Africa by comparing the genome of SARS-CoV-2 isolated from KwaZulu-Natal, South Africa with those isolated from China, Spain, Italy and United States, as well as the genomes of Bat SARS CoV, Middle East Respiratory Syndrome Coronavirus (MERS-CoV), Mouse Hepatitis Virus (MHV), and Infectious Bronchitis Virus (IBV). The study also investigated the ability of identified compounds from *C. sativa* to interact with gene segments (mRNAs) coding for proteins implicated in viral replication, assembly and release vis-à-vis ORF1ab, Surface glycoprotein, Envelope protein and Nucleocapsid phosphoprotein from the whole genome of SARS-CoV-2 isolated from KwaZulu-Natal, South Africa using computational tools.

## Materials and Methods

### Viral Genome Sequences

Different complete genome sequences (MT324062, MT291830, MT198652, MT077125, MT371047, FJ588686.1, MG923481.1, MF618252.1, and NC_001451.1) of SARS-CoV-2 (KwaZulu-Natal, South Africa), SARS-CoV-2 (Wuhan, China), SARS-CoV-2 (Valencia, Spain), SARS-CoV-2 (Rome, Italy), SARS-CoV-2 (New York, United States), Bat SARS CoV Rs672/2006, MERS-CoV, MHV and IBV respectively (Table 1), were retrieved from the National Center for Biotechnology Information (NCBI) database and thereafter subjected to alignment using CLUSTALW X Software, version 10.1.8 (Kumar et al., 2018) (Supplementary Figure S1). The aligned sequences were then used to plot phylogenetic tree.

**TABLE 1:** Viral genome codes used for the present study.

NCBI Genome Codes	Virus	Country Source
MT324062	SARS-CoV-2	South Africa (KwaZulu-Natal)
MT291830	SARS-CoV-2	China (Wuhan)
MT198652	SARS-CoV-2	Spain (Valencia)
MT077125	SARS-CoV-2	Italy (Rome)
MT371047	SARS-CoV-2	United States (NY)
FJ588686.1	Bat SARS CoV Rs672/2006	
MG923481.1	MERS-CoV	
MF618252.1	MHV	
NC_001451.1	IBV	

### Phylogenetic Analysis; Evolutionary Relationships of Taxa

The evolutionary history was inferred using the UPGMA method (Sneath and Sokal, 1973). The optimal tree with the sum of branch length = 1.90049281 is shown. The tree was drawn to scale, with branch lengths in the same units as those of the evolutionary distances used to infer the phylogenetic tree. The evolutionary distances were computed using the Maximum Composite Likelihood method (Tamura et al., 2004) and are in the units of the number of base substitutions per site. This analysis involved 9 nucleotide sequences of the aforementioned viruses. All ambiguous positions were removed for each sequence pair (pairwise deletion option). There was a total of 31,304 positions in the final dataset. Evolutionary analyses were conducted using MEGA X (Kumar et al., 2018).

### Plant Material

The use of *C. sativa* was approved by the South African Health Products Regulatory Authority to conduct, collect, posses, transport and store cannabis plant, plant parts and products for research purposes (Permit No. POS 248/2019/2020; permit issued to the University of Free State).

*Cannabis sativa* leaves were collected from Mohale’s Hoek District, Lesotho (GPS coordinates: 30.333776″S and 27.651201″E) under the permit (Permit #: 01/LS/2019/10/02–01). The leaves were deposited with the voucher number: BLFU MGM 0018 following its identification and authentication by the Geo Potts Herbarium at the University of the Free State, Bloemfontein 9300, South Africa.

### Infusion of *C. sativa* Leaves

The leaves were air-dried and blended to smooth powder. About 30 g of the powdered samples were infused in boiled water and allowed to extract for 2 h. The infusion was decanted into plastic bowls and allowed to freeze at −80°C before freeze-drying to yield about 9 g of concentrated infusion. The sample was stored in glass vials and stored at 2°C until further analysis.

### Gas Chromatography-Mass Spectrometric Analysis

The concentrated sample was subjected to GC-MS analysis in order to identify the compounds. This was carried out with an Agilent technologies 6890 Series GC coupled with (an Agilent) 5973 Mass Selective detector, which is driven by Agilent Chemstation software. The operating parameters were:

Column: HP-5MS capillary column (30 × 0.25 mm ID, 0.25 μm film thickness, 5% phenylmethylsiloxane); Carrier gas: ultra-pure helium; Gas flow rate: 1.0 ml/min at a linear velocity of 37 cm/s; Programmed oven temperature: 280°C (at the rate of 10 °C/min with a hold time of 3 min); Injector temperature: 250°C; Ion source temperature: 230°C; Quadrupole temperature: 150°C; Electron ionization mode: 70 eV; Electron multiplier voltage: 1859 V; Solvent delay: 4 min; Scan range: 50–70 amu. An inbuilt NIST data library was used in identifying the compounds via comparison of retention time and mass spectral data. The GC-MS spectra is shown in Supplementary Figure S2

### SARS-CoV-2 Protein mRNA Sequence

SARS-CoV-2 (KwaZulu-Natal, South Africa) whole genome mRNA sequence was obtained from PubMed database from corona virus whole genome (MT324062.1). Gene segment coding for ORF1ab, Surface glycoprotein, Envelope protein and Nucleocapsid phosphoprotein were identified. Possible initiation and termination sequence of the gene were selected based on the identification of regions carrying initiation and termination codons as shown in Table 2. About 45–50 nucleotide sequence from both initiation and termination codons were converted into 3D single mRNA strand using Discovery studio (v19.1.0.18287) and saved as protein data bank file (Biovia, 2015). The structures were prepared by removing all solvent molecules and optimized to simulate physiological conditions using Chimera v 1.1. Polar hydrogens were added, and partial charges were assigned to the standard residue using Gasteiger partial charge.

**TABLE 2:** Selected sequence of initiation and termination sites of target protein mRNAs from SARS-CoV-2 whole genome.

Gene Segment	Sites	Sequence
ORF1ab (266–21,555)	Translation initiation site	5′-AUG​GAG​AGC​CUU​GUC​CCU​GGU​UUC​AAC​GAG​AAA​ACA​CAC​GUC​CAA-3′
	Translation termination site	5′-AAC​AAC​AGA​GUU​GUU​AUU​UCU​AGU​GAU​GUU​CUU​GUU​AAC​AAC​UAA-3′
Surface glycoprotein (21,563–25,384)	Translation initiation site	5′- AUG​UUU​GUU​UUU​CUU​GUU​UUA​UUG​CCA​CUA​GUU​UCU​AGU​CAG​UGU​GUU-3′
	Translation termination site	5′- ACU​CUG​AGC​CAG​UGC​UCA​AAG​GAG​UCA​AAU​UAC​AUU​ACA​CAU​AA-3′
Envelope protein (26,245–26,472)	Translation initiation site	5′- AUG​UAC​UCA​UUC​GUU​UCG​GAA​GAG​ACA​GGU​ACG​UUA​AUA​GUU​AAU​A-3′
	Translation termination site	5′- AAA​AAT​CTG​AAT​TCT​TCT​AGA​GTT​CCT​GAT​CTT​CTG​GTC​TAA-3′
Nucleocapsid phosphoprotein (28,274–29,533)	Translation initiation site	5′- AUG​UCU​GAU​AAU​GGA​CCC​CAA​AAU​CAG​CGA​AAU​GCA​CCC​CGC​AUU​AC-3′
	Translation termination site	5′- AUU​GCA​ACA​AUC​CAU​GAG​CAG​UGC​UGA​CUC​AAC​UCA​GGC​CUA​A-3′

### Molecular Docking

The chemical structure of tetrahydrocannabivarin, cannabispiran, cannabidiol tetrahydrocannabinol, cannabigerol, cannabinol, hydroxychloroquine and remdesivir were retrieved from PubChem online webserver. Gaussian 09 equipped with Gaussview 5.0 was used to optimize the chemical structures (ligands). The optimize ligands were then converted to PDB files using UCSF Chimera tool. The crystal structure of the mRNA (receptors) was built from selected sequence of initiation and termination sites obtained from SARS-COV 2 proteins by making use of UCSF Chimera tool. Hydrogen and Gasteiger partial charges were added to the receptor using the UCSF Chimera tool. Molecular docking simulation was performed on a nucleic acid docking server (HNADOCK). Finally, the ligand-mRNA interaction was assessed using Maestro 11.5 a Schrodinger 2018–1 suite package.

Hydroxychloroquine and remdesivir were chosen as the control antiviral drugs owing to previous reports on their use in the treatment and management of COVID-19 (Al-Tannak et al., 2020; Wang et al., 2020).

## Results

### Phylogenetic Analysis

**FIGURE 1:**
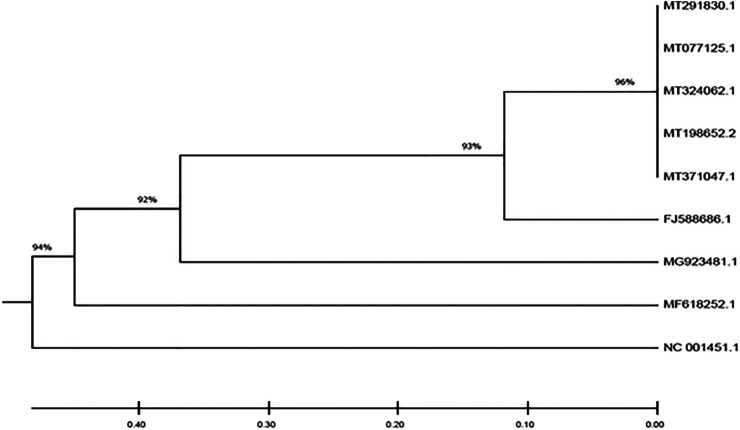
Phylogenetic tree depicting the evolutionary relationships between the studied viral genomes.

The South African SARS-CoV-2 isolate evidently clustered with other SARS-CoV-2 isolates from other countries (China, Italy, United States and Spain) with 96% homology as shown in Figure 1. Interestingly, it also showed 93% homology with the isolate from Bat. This was followed by 92 and 94% homology with MERS-CoV isolate and MHV/IBV, respectively.

### Gas Chromatography-Mass Spectrometric Analysis

**FIGURE 2:**
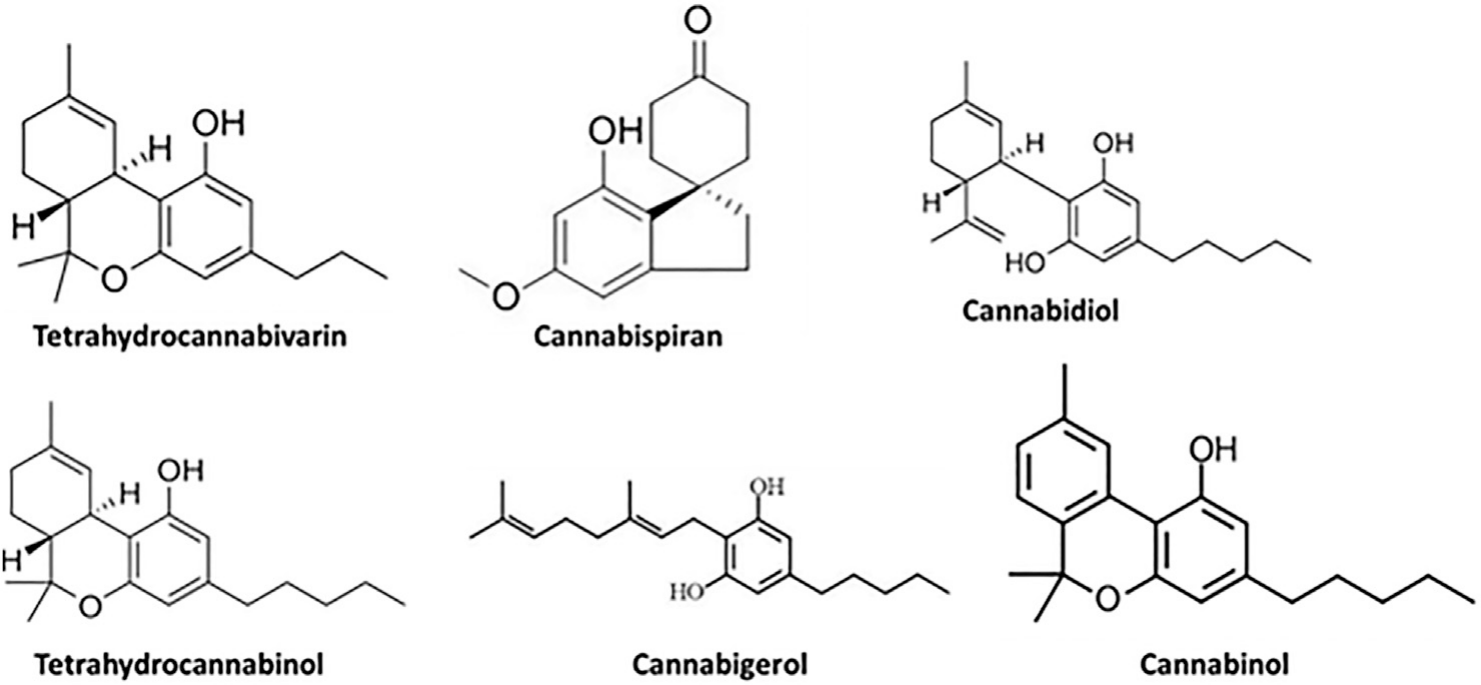
GC-MS identified compounds from *C. sativa* infusion.

As depicted in Figure 2 and Table 3, GC-MS analysis of the infusion revealed the presence of tetrahydrocannabivarin, cannabispiran, CBD, THC, cannabigerol and cannabinol. The highest concentration were THC and cannabinol, with 73.84 and 21.74% abundance, respectively as shown in Table 3.

**TABLE 3:** GC-MS identified compounds in *C. sativa* infusion.

Compounds	% Abundance	Molecular formular	Molecular weight
Tetrahydrocannabivarin	1.66	C_19_H_26_O_2_	286.4
Cannabispiran	0.07	C_15_H_18_O_3_	246.3
Cannabidiol	1.67	C_21_H_30_O_2_	314.5
Tetrahydrocannabinol	73.84	C_21_H_30_O_2_	314.5
Cannabigerol	1.03	C_21_H_32_O_2_	316.5
Cannabinol	21.74	C_21_H_26_O_2_	310.4

### Molecular Docking

**FIGURE 3:**
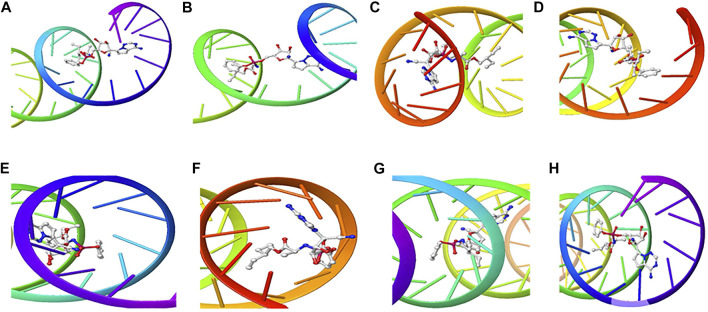
3D Interaction of Remdesivir with Initiation and Termination Sequence of the Viral Proteins. A = Nucleocapsid Phosphoprotein Translation Initiation Site; B = Envelope Protein Translation Initiation Site; C = Envelope Protein Translation Termination Site; D = Nucleocapsid Phosphoprotein Translation Termination Site; E = ORF1ab Translation Initiation Site; F = ORF1ab Translation Termination Site; G = Surface Glycoprotein Translation Termination Site; H = Surface Glycoprotein Translation Initiation Site.

**FIGURE 4:**
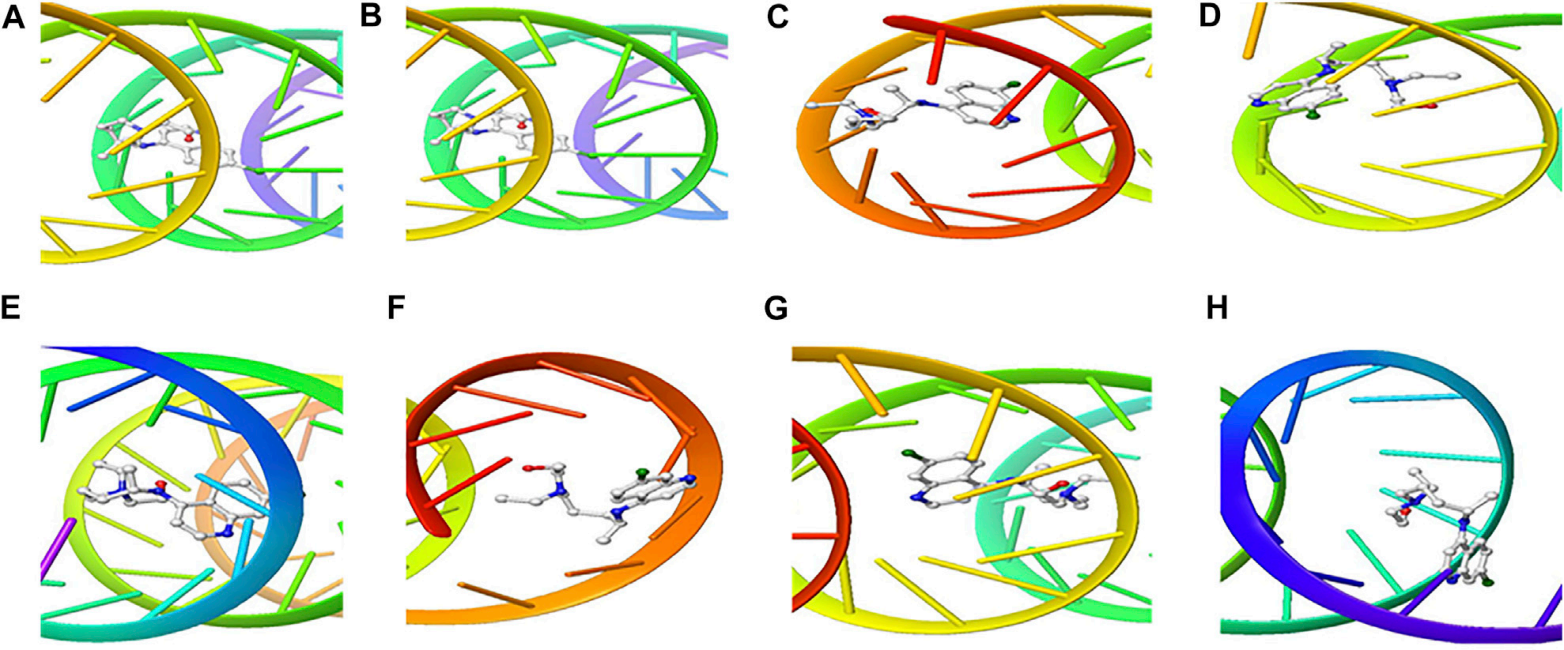
3D Interaction of hydroxychloroquine with Initiation and Termination Sequence of the Viral Proteins. A = Nucleocapsid Phosphoprotein Translation Initiation Site; B = Envelope Protein Translation Initiation Site; C = Envelope Protein Translation Termination Site; D = Nucleocapsid Phosphoprotein Translation Termination Site; E = ORF1ab Translation Initiation Site; F = ORF1ab Translation Termination Site; G = Surface Glycoprotein Translation Termination Site; H = Surface Glycoprotein Translation Initiation Site.

**FIGURE 5:**
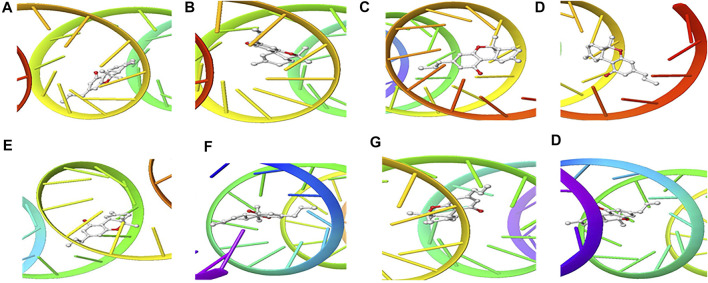
3D Interaction of tetraydrocannabivarin with Initiation and Termination Sequence of the Viral Proteins. A = Nucleocapsid Phosphoprotein Translation Initiation Site; B = Envelope Protein Translation Initiation Site; C = Envelope Protein Translation Termination Site; D = Nucleocapsid Phosphoprotein Translation Termination Site; E = ORF1ab Translation Initiation Site; F = ORF1ab Translation Termination Site; G = Surface Glycoprotein Translation Termination Site; H = Surface Glycoprotein Translation Initiation Site.

**FIGURE 6:**
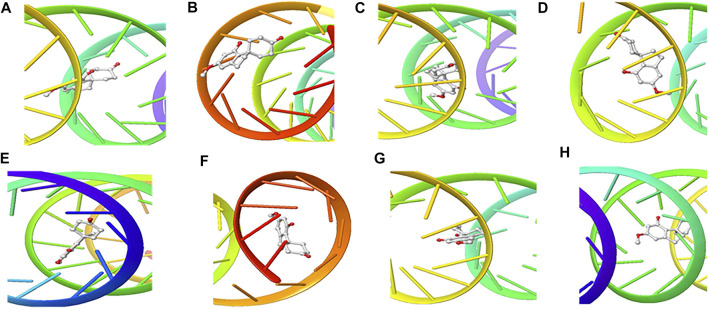
3D Interaction of Cannabispiran with Initiation and Termination Sequence of the Viral Proteins. A = Nucleocapsid Phosphoprotein Translation Initiation Site; B = Envelope Protein Translation Initiation Site; C = Envelope Protein Translation Termination Site; D = Nucleocapsid Phosphoprotein Translation Termination Site; E = ORF1ab Translation Initiation Site; F = ORF1ab Translation Termination Site; G = Surface Glycoprotein Translation Termination Site; H = Surface Glycoprotein Translation Initiation Site.

**FIGURE 7:**
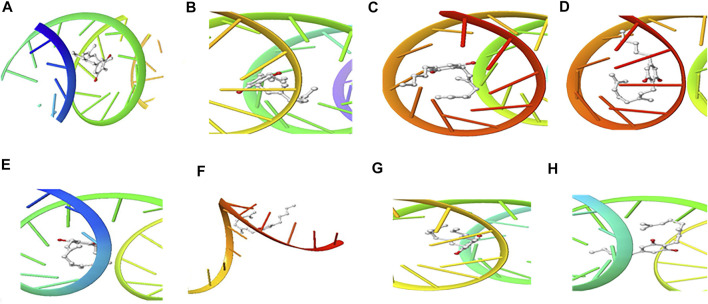
3D Interaction of Cannabigerol with Initiation and Termination Sequence of the Viral Proteins. A = Nucleocapsid Phosphoprotein Translation Initiation Site; B = Envelope Protein Translation Initiation Site; C = Envelope Protein Translation Termination Site; D = Nucleocapsid Phosphoprotein Translation Termination Site; E = ORF1ab Translation Initiation Site; F = ORF1ab Translation Termination Site; G = Surface Glycoprotein Translation Termination Site; H = Surface Glycoprotein Translation Initiation Site.

**FIGURE 8:**
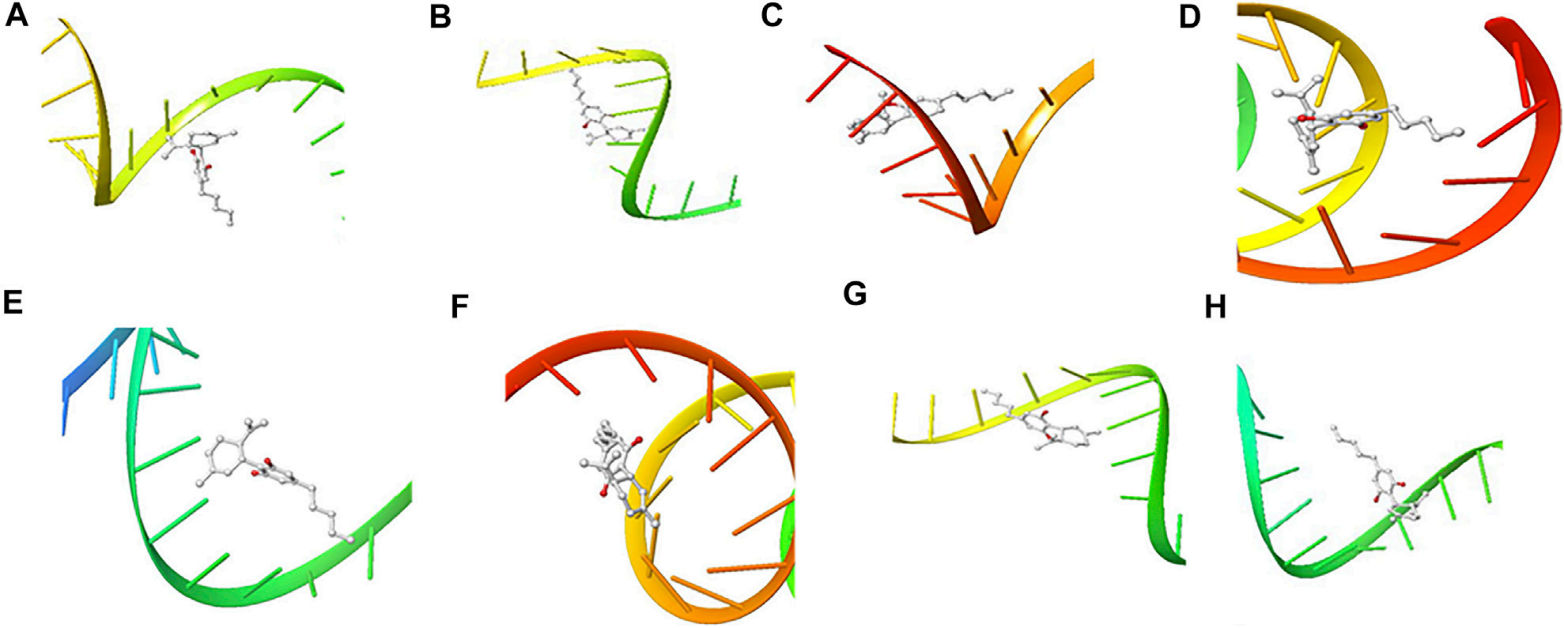
3D Interaction of Cannabidiol with Initiation and Termination Sequence of the Viral Proteins. A = Nucleocapsid Phosphoprotein Translation Initiation Site; B = Envelope Protein Translation Initiation Site; C = Envelope Protein Translation Termination Site; D = Nucleocapsid Phosphoprotein Translation Termination Site; E = ORF1ab Translation Initiation Site; F = ORF1ab Translation Termination Site; G = Surface Glycoprotein Translation Termination Site; H = Surface Glycoprotein Translation Initiation Site.

The structure of the compounds and their best docked conformations within the binding site of the selected receptors are shown in Figures 3–8. These favorable interactions between the molecules are depicted by the binding energy as indicated in Table 4. All the compounds bounded to the target sequence reasonably with similar binding pattern. The bindings were seen within the beginning, middle and end of the initiation and termination codons of the various viral proteins respectively. These interactions include both polar and nonpolar through hydrogen, hydrophobic and other non-conventional interactions.

**TABLE 4:** Docking scores (Kcal^−1^) of molecular docking studies.

RCP	Cannabinol	Tetraydroconnabiran	Tetrahydrocannabinol	Cannabispiran	Hydroxychloroquine	Remdesivir	Cannabidiol	Cannabigerol
(Kcal^−1^)								
EPIS	−37.68	−39.11	−37.79	−37.43	−63.35	−86.81	−37.00	−39.53
EPST	−36.95	−36.93	−39.90	−36.95	−64.83	−88.47	−35.70	−37.92
NIS	−38.58	−39.18	−37.74	−39.29	−67.16	−92.05	−38.95	−38.36
NST	−38.14	−40.61	−40.68	−36.21	−63.11	−87.16	−37.24	−40.78
OLIS	−37.29	−41.61	−41.62	−41.36	−69.71	−89.52	−39.71	−40.51
OLST	−38.39	−39.97	−39.54	−37.80	−64.10	−90.18	−38.20	−39.62
SGIS	−33.77	−35.09	−34.25	−33.81	−54.72	−81.52	−32.01	−35.36
SGST	−39.65	−40.99	−41.19	−41.00	−65.92	−90.76	−39.68	−40.56

**RCP:** Receptors; EPIS = Envelope protein Translation initiation site; EPST = Envelope protein Translation termination site; NIS = Nucleocapsid phosphoprotein Translation initiation site; NST = Nucleocapsid phosphoprotein Translation termination site; OLIS = ORF1ab Translation initiation site; OLIST = ORF1ab Translation termination site; SGIS = Surface glycoprotein Translation initiation site; SGST = Surface glycoprotein Translation termination site.

## Discussion

Since its outbreak in Wuhan China, SARS-CoV-2 has spread across the globe leading to the COVID-19 pandemic. The spread of the disease has been attributed mostly to infected travelers who act as carrier of the virus, and then leading to community spread. In the present study, we decoded the origin of the virus in South Africa by comparing the genome of SARS-CoV-2 isolated from KwaZulu-Natal, South Africa with those of countries with reported high cases (Table 1). The 96% homology with genomes from China, Spain Italy and United States suggests SARS-CoV-2 in South Africa originated from outside the continent and corroborates reports that her index COVID-19 case was a male citizen who tested positive to the virus after a trip to Italy (SAG, 2020). This also corroborates the global spread of the virus and the disease (Lee, 2020; Macintyre, 2020). Furthermore, the 93% homology of the genome of the South African isolate with bat SARS COVs, as well as the 92 and 94% homology with MERS-COV and MHV/IBV isolates, respectively insinuates an evolutionary conservation among the whole genomes of the studied coronaviruses. These evolutionary trends corroborate reports that suggest that coronaviruses are of bat origin (Lu et al., 2020; York, 2020; Zhou et al., 2020).

Coronaviruses are enveloped viruses with a single strand, positive-sense RNA genome of 27–32 kb in length (Liu et al., 2007). Several studies have reported more than 100 full-length or partial genomic sequences for SARS-COV-2 (Wang et al., 2020). The genome contains about six to nine sorts of mRNA, comprising the genome-length mRNA and five to eight sub-genomic mRNA which code for structural protein and many other for non-structural proteins (Liu et al., 2007). In an infected cell, coronaviruses induce the formation of double-membrane vesicles and convoluted membranes that harbor the nonstructural proteins (Verheije et al., 2010). The structural proteins include transmembrane spike glycoprotein, nucleocapsid phosphoprotein, envelope, and surface glycoproteins (Baudoux et al., 1998), whereas the nonstructural includes ORF1ab, ORF3, ORF6, ORF7a, ORF8, and ORF10 which contains information for genome RNA synthesis and replication (Tsai et al., 2020). The entry mechanism of SARS-CoV-2 starts with its transmembrane spike glycoprotein attachment to host cell membrane receptor (angiotensin-converting enzyme 2) and induce its entry through membrane endocytosis. ORF1 stimulates the virus replication and synthesis of the subgenomic RNAs, while nucleocapsid phosphoprotein packages the viral genome to form the helical nucleocapsid that is unified into the budding particle and can also function as an RNA chaperone. Envelope and surface glycoproteins induce virion assembly and morphogenesis leading to the formation of virus-like particles and the final release of the virions by exocytosis (Ho et al., 2004; Zúñiga et al., 2007; Andersen et al., 2020). Any attempt to interfere with the expression of one of these proteins could be tantamount to disrupting the overall SARS-COV-2 transmission cycle. Therefore, targeting these structural and nonstructural protein components especially at translational level of mRNAs being converted to proteins, could serve as a viable therapeutic strategy against SARS-COV-2. This is corroborated by studies which reported ORF1ab, surface glycoprotein, nucleocapsid phosphoprotein, envelope, membrane, spike protein, protease, hemagglutinin esterase and helicase as possible therapeutic target for coronaviruses including SARS-CoV-2 (Mcbride et al., 2014; Bhatia et al., 2020). The strong binding affinities of the identified compounds from *C. sativa* (Figure 2 and Table 3) with both the initiation and termination codons of ORF1ab, Surface glycoprotein, Envelope protein and Nucleocapsid phosphoprotein mRNAs from the whole genome of SARS-CoV-2 isolated from KwaZulu-Natal, South Africa (Figures 3–8) therefore, portray their high potential as possible therapy for treatment of COVID-19 and its complication by curbing the replication, translation, assembly, and release of the virus. The mechanism of these strong binding affinities of the identified compounds from *C. sativa* againts the identified mRNA initiation and termination codons could be due some chemico-biological interactions based on the chemistry of the said bioactive compounds versus that of nucleotides. Literally, the constituents of mRNA include adenine, guanine, cytosine, uracil and a phosphate terminal (Elliott and Ladomery, 2017). Meanwhile, these chemical compounds are classified as purine and pyrimidines and are known to constitute basic functional group (amine and carbonyl) with the capacity to undergo nucleophilic or electrophilic reactions (Ulbricht, 2016). In the biological system, natural product dissociates, hence, the tendency to interact with purines and pyrimidines via electrostatic, hydrophobic, intermolecular force or covalent bond is certain. GC-MS analysis of *Cannabis sativa* extract revealed the presence of tetrahydrocannabivarin, cannabispiran, cannabidiol tetrahydrocannabinol, cannabigerol, and cannabinol. These compounds contain free hydroxyl and aromatic rings which confers on them the ease to interact with the purines, pyrimidines, or phosphate terminal of SARS-CoV-2 genome mRNA sequence via hydrogen bonding or intermolecular interaction. Hydrogen bond formation between the oxygen at carbon 2 of the purines on the genome of SARS-CoV-2 mRNA may be responsible for its interaction with natural product. On the other hand, the free amine attached to carbon 2 and 6 of guanine and adenine respectively may account for the mechanism of interactions (electrostatic) between pyrimidines and the natural product obtained from *Cannabis sativa* extract. Similarly, the phosphate terminal of the SARS-CoV-2 mRNA could electrostatically interact with the polar region of electron deficient natural product that may have dissociated with the biochemical environment. Hence, at a favourable pH within the biochemical system, natural product obtained from *Cannabis sativa* extract can dissociate to impart a possible therapeutic function against COVID-19. Furthermore, flavonoid-based molecules from *C. sativa* have also been shown to bind with high affinity to the spike protein, helicase, and protease sites on the ACE2 receptor used by the coronavirus 2 to infect cells and cause COVID-19 (Attia et al., 2020; Khanna et al., 2020).

Interestingly, the viral proteins have also been implicated in manipulating the host’s innate immune system (Alexander et al., 2019). Conversely, *C. sativa* and its phytocannabinoids have been proposed as a possible natural products for the treatment of COVID-19 (El Biali et al., 2020; Mckernan et al., 2020). This is also coupled with their reported potent anti-inflammatory activities esepecially CBD, tetrahydrocannabivarin and THC (Bolognini et al., 2010; Anil et al., 2020; Nichols and Kaplan, 2020) as the pathologenesis and progression of the virus have been linked to excessive producition of pro-inflammatory cytokines often termed as cytokine storm syndrome leading to suppression of the immune system (Ragab et al., 2020; Vaninov, 2020).

These results further indicate that phytochemicals from *C. sativa* can respond to the COVID-19 at different levels of its mRNAs and different mechanisms. They corroborate previous studies that tentatively indicated that CBD and THC may be beneficial in the treatment of patients whose bodies’ inflammatory response has become pathogenic and therefore respond to the cytokine storm (Nagarkatti et al., 2009; Onaivi and Sharma, 2020; Rossi et al., 2020). Much research in this field has focused on the ability of cannabinoids and terpenes to lower the immune system’s response without suppressing.

## Conclusion

Taken together, the results from this study indicates a homology between the genome of SARS-CoV-2 isolated from KwaZulu-Natal, South Africa and those isolated from Europe, Asia and North America, as well as those isolated from bat SARS COV, MERS-CoV, MHV and IBV. Thus, depicting the spread of the virus is intercontinental and might have evolved from other coronaviruses. The results also indicate the phytocannabinoids of *C. sativa* infusion as potential therapies against COVID-19 as depicted by their ability to molecularly interact with codon mRNAs of proteins implicated in the replication, translation, assembly, and release of SARS-CoV-2. However, further studies are needed to verify these activities pre-clinically and clinically. It is also recommended that LC-MS analysis should be carried out on the infusion to further identify its polar constituents.

## Data Availability

The original contributions presented in the study are included in the article/Supplementary Material, further inquiries can be directed to the corresponding author.
