# Supplementary material for: Targeting of Protein’s Messenger RNA for Viral Replication, Assembly and Release in SARS-CoV-2 Using Whole Genomic Data From South Africa: Therapeutic Potentials of Cannabis Sativa L
Source: Front Pharmacol. 2021 Sep 2;12:736511. doi: 10.3389/fphar.2021.736511 (PMC8448283; doi:10.3389/fphar.2021.736511)
Supplement: Supplementary file 1 [file DataSheet1.docx]

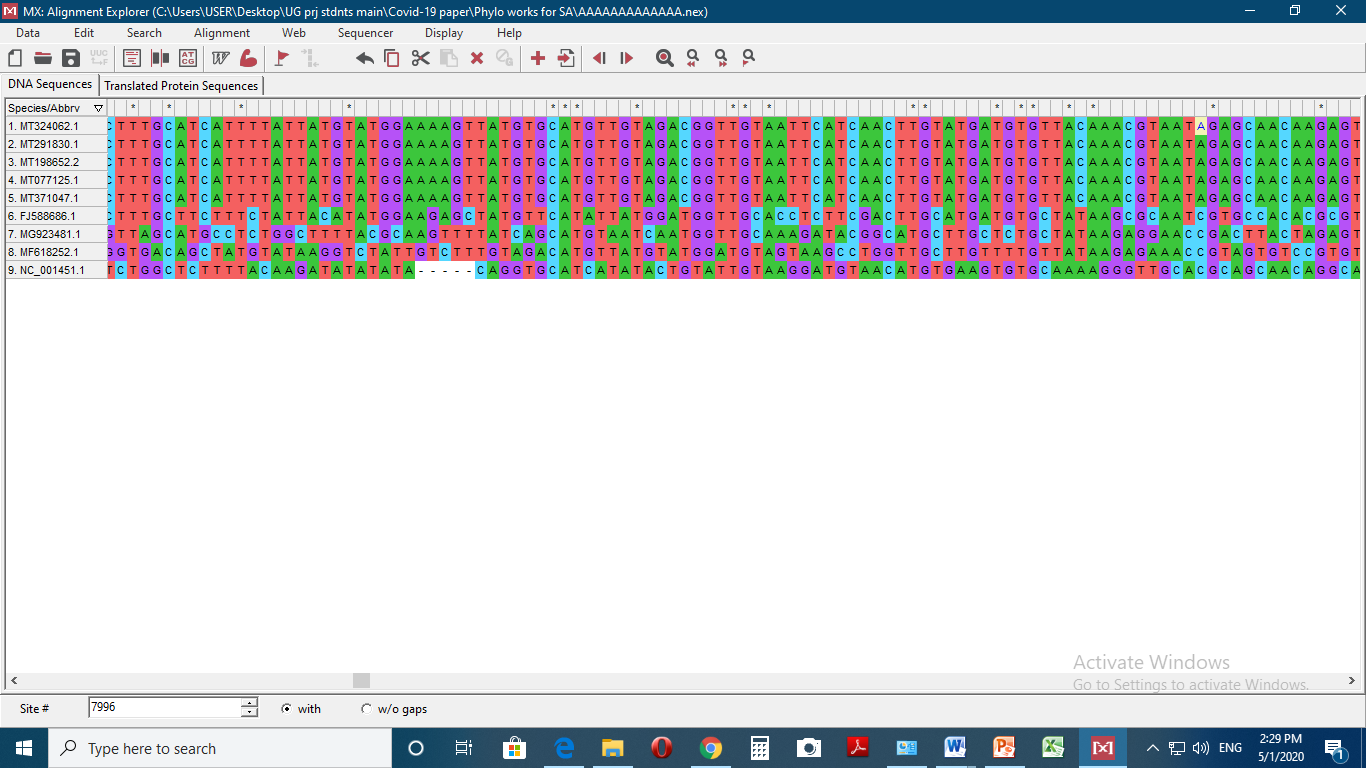


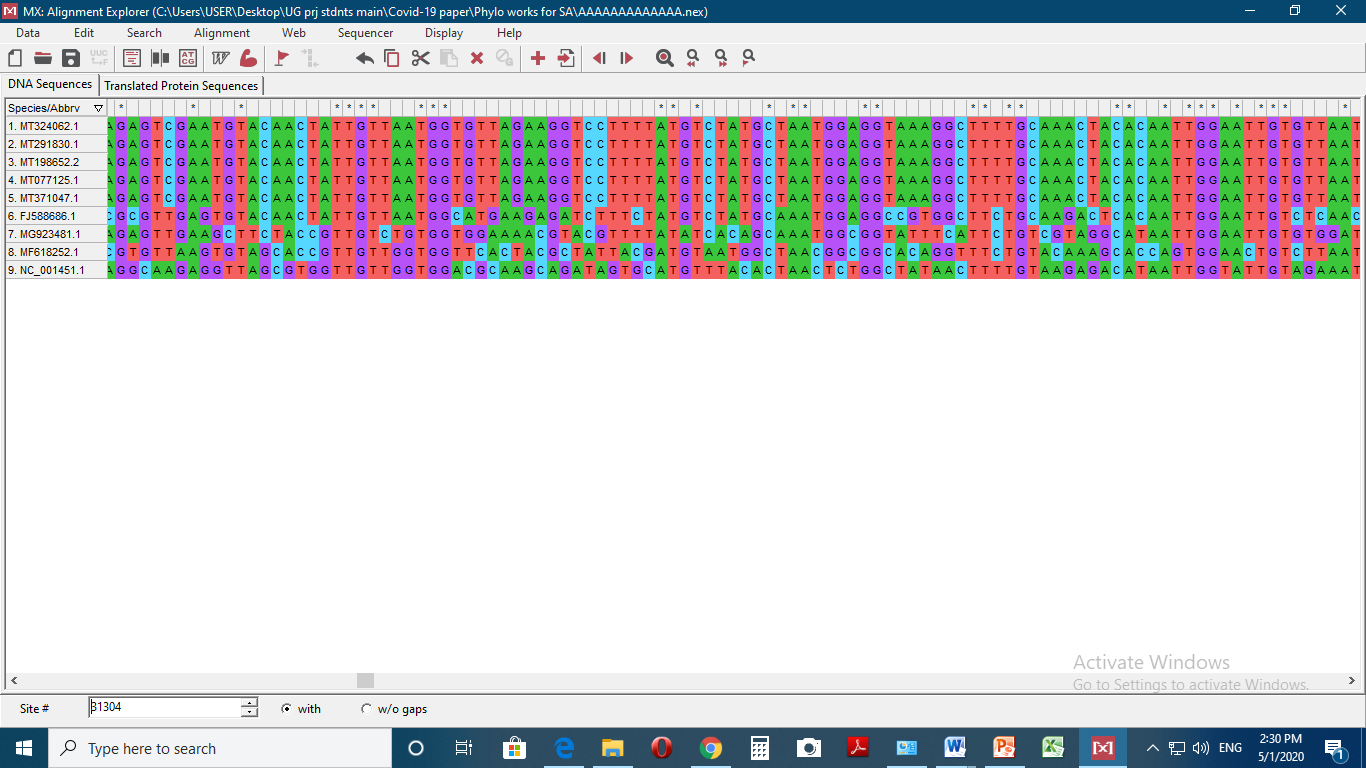


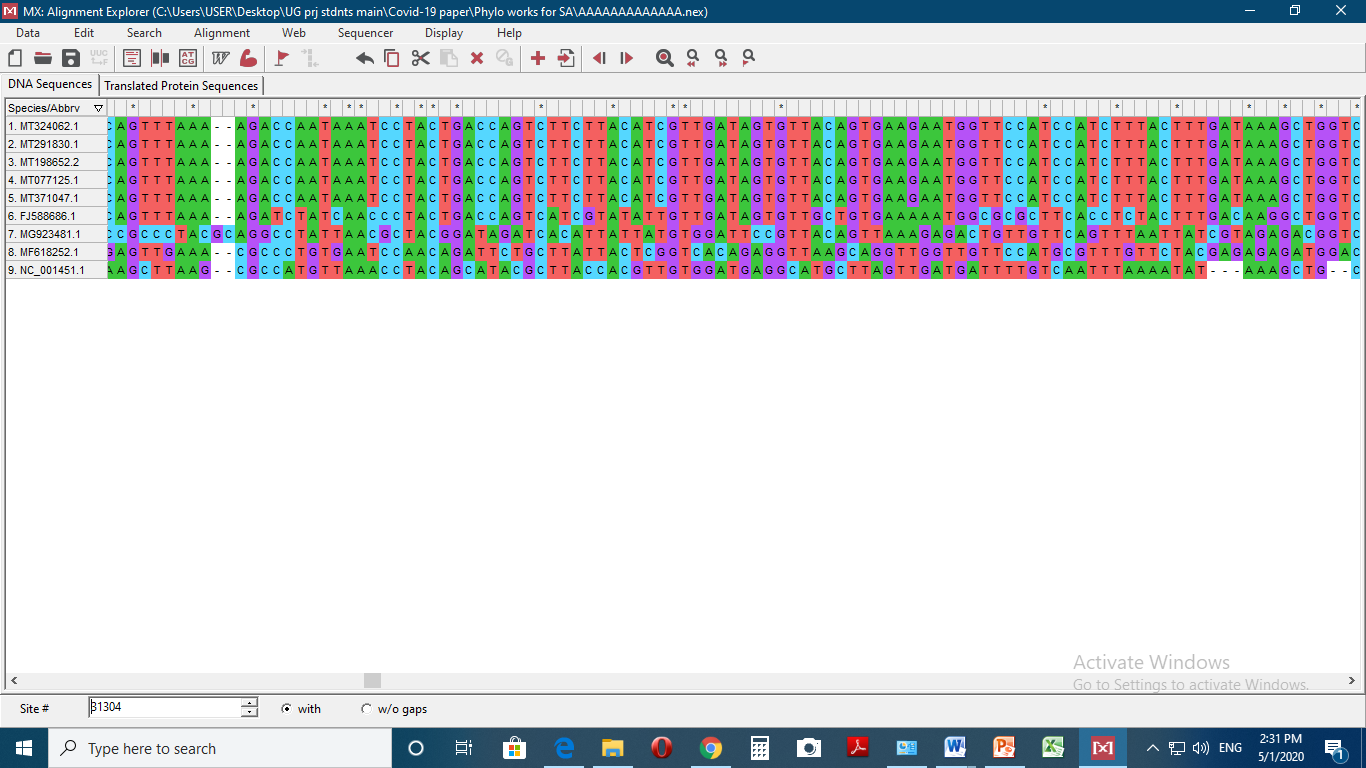


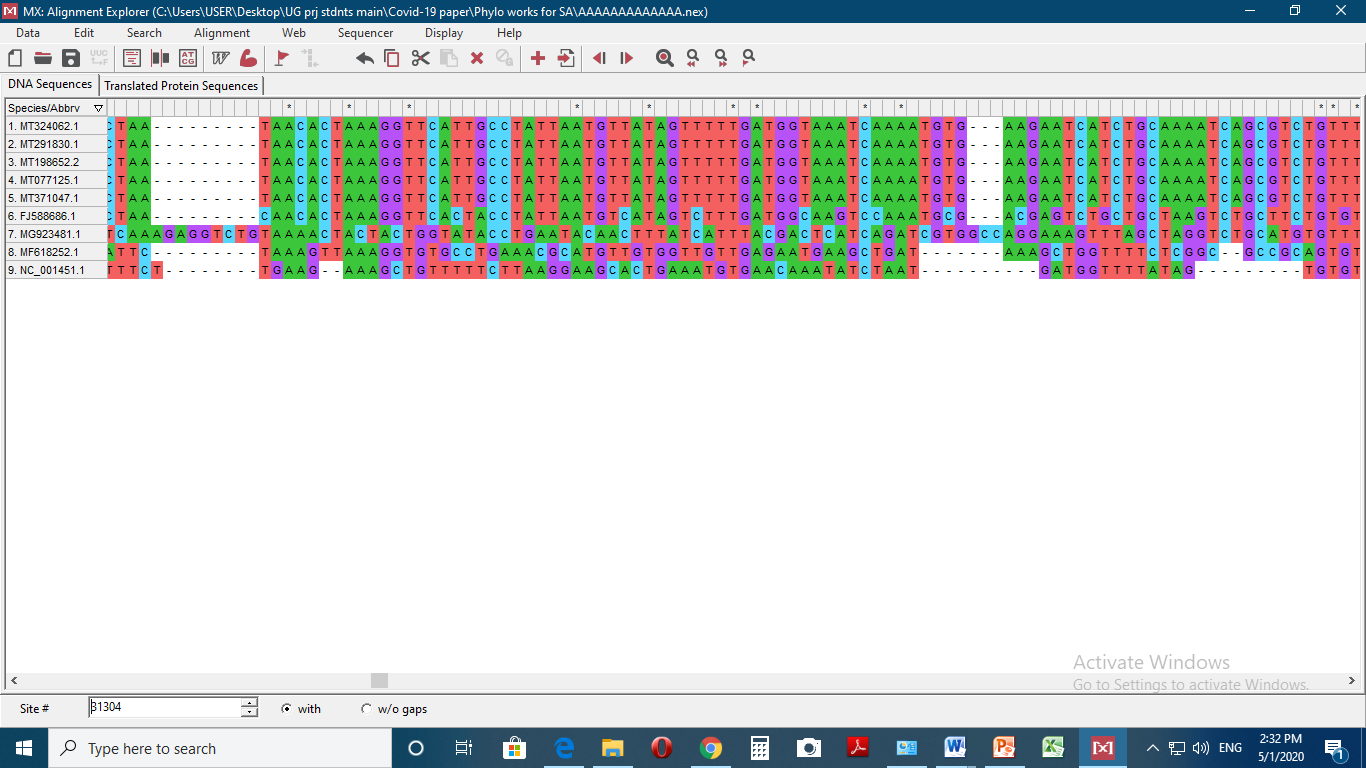


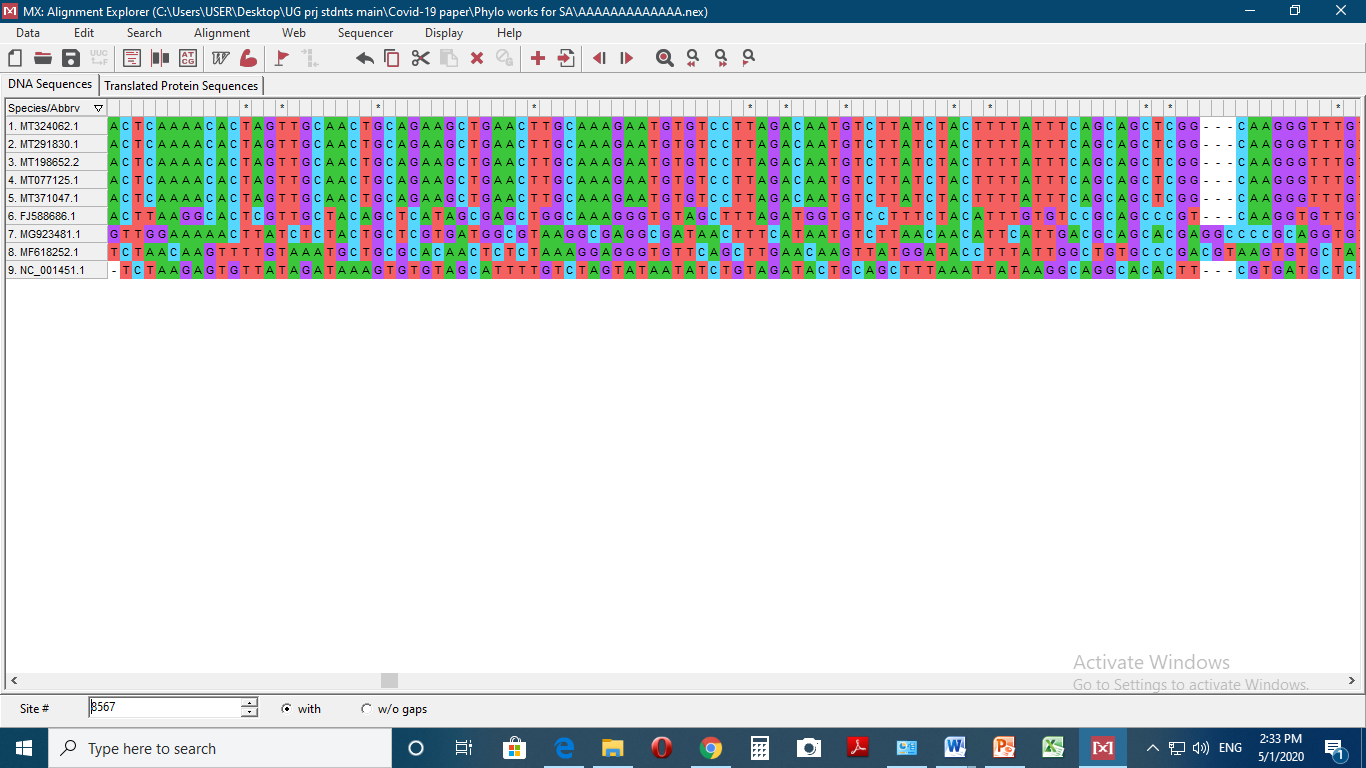


Fig. S1: Multiple sequence alignment of different conserved regions of studied viral genomes

Fig. S2: GC-MS spectra of identified compounds in *C. sativa* infusion
